# Wnt10b regulates osteogenesis of adipose‐derived stem cells through Wnt/β‐catenin signalling pathway in osteoporosis

**DOI:** 10.1111/cpr.13522

**Published:** 2023-06-20

**Authors:** Kui Huang, Shuyu Cai, Ting Fu, Qiang Zhu, Lin Liu, Zhihao Yao, Pengcheng Rao, Xiaorong Lan, Qing Li, Jingang Xiao

**Affiliations:** ^1^ Department of Oral and Maxillofacial Surgery The Affiliated Stomatological Hospital of Southwest Medical University Luzhou China; ^2^ Luzhou Key Laboratory of Oral & Maxillofacial Reconstruction and Regeneration Luzhou China; ^3^ Department of Oral Implantology The Affiliated Stomatological Hospital of Southwest Medical University Luzhou China; ^4^ Department of Oral and Maxillofacial Surgery The Affiliated Hospital of Southwest Medical University Luzhou China

## Abstract

Our previous finding revealed that the *Wnt10b* RNA expression of osteoporotic adipose‐derived stem cells (OP‐ASCs) with impaired osteogenic capacity was significantly reduced than that of ASCs. There are no ideas that the relationship between the OP‐ASCs' impaired osteogenic potential and *Wnt10b* expression. This study aimed to indicate the potential molecular mechanisms and functional role of *Wnt10b* in OP‐ASCs, as well as to investigate a potential application to reverse the OP‐ASCs' impaired osteogenic differentiation potential. The OP‐ASCs and ASCs were harvested from the inguinal fat of osteoporosis (OP) mice with bilateral ovariectomy (OVX) and normal mice. qPCR and WB were used to detect the different levels of the expression of the *Wnt10b* RNA in both OP‐ASCs and ASCs. Lentiviral‐mediated regulation of *Wnt10b* expression was employed for OP‐ASCs, and the detection of the expression levels of key molecules in the Wnt signalling pathway and key osteogenic factors was performed through qPCR and WB in vitro experiments. The capacity of OP‐ASCs to osteogenesis was determined using alizarin red staining. Lastly, the repair effect of the BCP scaffolds incorporating modified OP‐ASCs on the critical‐sized calvarial defects (CSCDs) in OP mice was scanned and detected by micro‐computed tomography, haematoxylin and eosin staining, Masson's trichrome staining and immunohistochemistry. First, we discovered that both the RNA and protein expression levels of *Wnt10b* were significantly lower in OP‐ASCs than that in ASCs. In vitro experiments, upregulation of Wnt10b could activate the Wnt signalling pathway, and increase expression of β‐catenin, Lef1, Runx2 and osteopontin (Opn), thereby enhancing the osteogenic ability of OP‐ASCs. In addition, the OP‐ASCs with *Wnt10b*‐overexpressing could promote the repair of CSCD in osteoporotic mice with increasing new bone volume, bone mineral density, and increased expression of Opn in new bone in vivo. Taken together, overexpression of *Wnt10b* could partially facilitate the differentiation of OP‐ASCs towards osteogenesis and accelerated the healing of bone defects by activating the Wnt/β‐catenin signalling pathway in vitro and in vivo experiments. This study confirmed the important role of *Wnt10b* in regulating the osteogenic differentiation capability of OP‐ASCs and indicated *Wnt10b* could be a potential therapeutic target for reversing the impaired osteogenic capabilities of OP‐ASCs to therapy bone defects of OP patients.

## Graphical abstract

In this study, osteoporotic adipose‐derived stem cells (OP‐ASCs) were successfully isolated and cultured from the inguinal fat of the OP mouse with bilateral ovariotomy. Lentiviral‐mediated overexpression of *Wnt10b* could enhance the osteogenic ability of OP‐ASCs by activating the canonical Wnt signalling pathway to promote the repair of critical‐sized calvarial defect in osteoporotic mice.

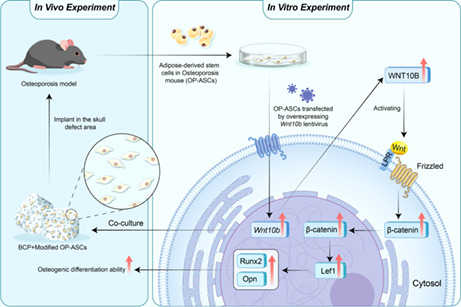

## 1 INTRODUCTION

Postmenopausal osteoporosis (OP) is a prevalent, devastating systemic and chronic disease. The main characteristics of postmenopausal OP are gradually compromised bone strength and reduced bone mineral density (BMD). Millions of women who suffered from postmenopausal OP are at a high risk of osteoporotic fractures and many related complications, and it remains a challenge to repair the subsequent bone defects.^1^ Although autografts with osteoinductive and osteoconductive are the gold standard in the clinic for the reparation and reconstruction of bone defects, their drawbacks, including pain, infection and restricted supply, remained unresolved.^2^ In recent years, with the rapid development of gene therapy and its associated materials, the technologies of tissue engineering have been regarded as promising therapeutic strategies in the treatment and reconstruction of bone defects.^3^, ^4^, ^5^ Seed cells used in tissue engineering, including ASCs, bone marrow‐derived mesenchymal stem cells (BMSCs), and muscle‐derived stem cells, have been engineered to overexpress factors to promote osteogenesis and angiogenesis for bone regeneration in bone defects.^6^, ^7^, ^8^ Among the seed cells, ASCs are increasingly preferred by researchers in bone regeneration and tissue engineering research because of their relative ease of access, high yield, ease of culture, rapid growth and resistance to ageing.^9^, ^10^, ^11^ Furthermore, ASCs have demonstrated similar osteogenic differentiation potential to BMSCs in vitro and in vivo,^12^ and some researchers believe that ASCs are superior to BMSCs in some ways.^13^ Recent study has shown that ASCs exhibit osteogenic potential in both orthotopic and ectopic sites.^14^ ASCs have been shown in studies to be more osteogenic than BMSCs in ovariectomized (OVX) rats.^15^ Compared with allogeneic OP‐ASCs, transplantation of autologous OP‐ASCs offers advantages in terms of stability, safety and portability in the treatment of OP‐related bone deformities, implying that treating OP and related bone defects with autologous OP‐ASCs may be a promising approach with potentially higher efficiency.^15^ Therefore, the use of autologous OP‐ASC transplantation offers a greater range of applications in the treatment of OP and associated bone defects.

**FIGURE 1:**
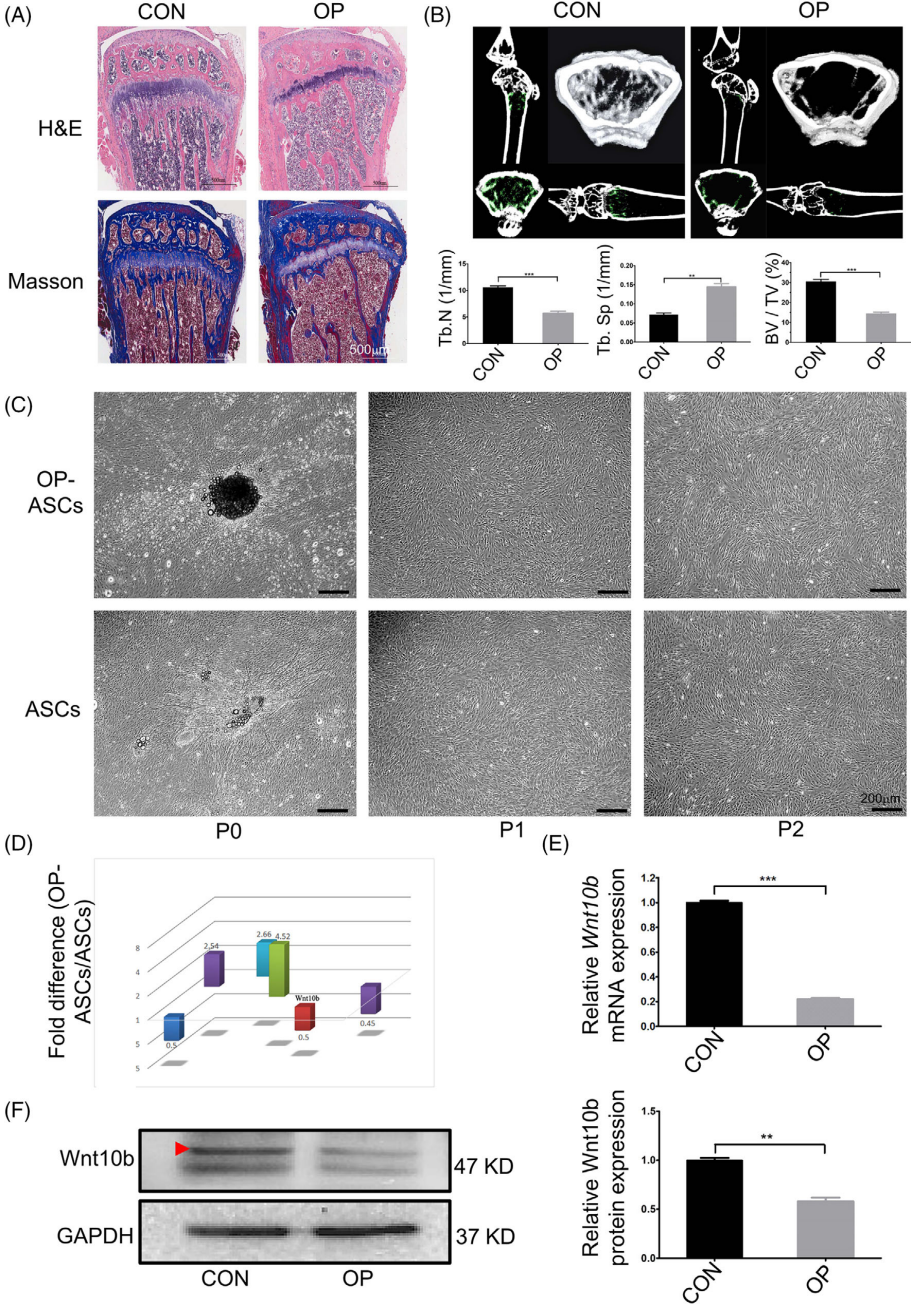
Wnt10b exhibited lower expression in OP‐ASCs than ASCs. (A) H&E and Masson's trichrome staining of the proximal metaphysis of the tibia from CON and OVX mice at 6‐week post‐operation. (B) The structure and morphology of the femur metaphysis from CON and OVX mice at 6‐week post‐operation obtained through micro‐CT, and statistical analysis of the Tb.N, BV/TV, and Tb.Sp between CON and OVX mice. (C) Images of OP‐ASCs and ASCs at different generations. (D) Expression of Wnt10b in OP‐ASCs was significantly lower than that observed in ASCs by GeneChip. (E) qPCR and statistical analysis showed that the mRNA expression level of Wnt10b was significantly decreased in OP‐ASCs when compared with ASCs. (F) Protein expression of Wnt10b in OP‐ASCs was lower than that in ASCs confirmed through WB. BV/TV, bone volume to tissue volume; CON, control; H&E, haematoxylin and eosin; micro‐CT, micro‐computed tomography; OP‐ASC, osteoporotic adipose‐derived stem cell; OVX, ovariectomized.

The Wnt pathway is thought to be an essential regulator of bone homeostasis.^16^, ^17^ and is composed of different ligands, coreceptors, receptors, and inhibitors. In a previous study, we performed GeneChip on OP‐ASCs and ASCs and found that compared with ASCs, the expression of the *Wnt10b* RNA was significantly reduced in OP‐ASCs (Figure 1D). Current studies on *Wnt10b* indicate that it acts as a candidate member of the Wnt family to regulate the fate of mesenchymal stem cells (MSCs) to promote osteogenesis, and it has been shown to be a significant endogenous regulator of bone formation by activating highly conserved Wnt signalling pathways. β‐catenin is essential for *Wnt10b*, Wnt10a and Wnt6 to regulate osteoblastogenesis and adipogenesis.^17^, ^18^ Thus, β‐catenin as a downstream factor is required for these Wnt ligands to regulate MSC fate.^19^ Therefore, *Wnt10b* is an important gene in the Wnt ligand family that encodes a secreted protein to regulate the bone metabolism. FABP4‐*Wnt10b* mice have been shown to have increased bone mass as well as higher levels of Wnt10b expression in the bone marrow. Additionally, *Wnt10b* knockout mice also show decreased osteocalcin levels and bone trabeculae.^20^ The mechanism is suspected to be related to a change of cell fate by promoting the transformation of mesenchymal precursor cells into osteoblastic phenotypes corresponding with inhibiting the differentiation towards adipocyte lines.^20^, ^21^ Co‐activation of endogenous Foxc2 and *Wnt10b* could enhance osteogenic differentiation of BMSCs and their differentiation towards lipogenesis, thereby improving the healing of calvarial bone defects in rats.^22^ However, there are few studies on the osteogenic ability of *Wnt10b* on ASCs and OP‐ASCs.

Biphasic calcium phosphate (BCP) ceramics, mainly composed of β‐tricalcium phosphate (β‐TCP) and hydroxyapatite (HA) in various ratios and have been marketed and accepted as bone replacement materials in many medical and dental applications.^23^ BCP is a material with osteoconductive capacity because of its unique interconnectivity and porosity, where better intercellular communication between osteogenic cells could take place.^23^, ^24^ In our study, BCP scaffolds were applied as cell carriers in vivo to explore the effects of osteogenesis.

To investigate underlying molecular mechanisms, explore the functional role of *Wnt10b* in OP‐ASCs, enhance OP‐ASC osteogenic capability and promote bone regeneration of osteoporotic patients with bone defects. In our past research, the expression profiles of OP‐ASCs and ASCs were determined by full transcriptome microarrays. We found that the *Wnt10b* mRNA expression was significantly decreased in OP‐ASCs compared with ASCs. Moreover, qPCR and WB were utilized to examine the *Wnt10b* RNA and protein expression differences between the two types of cells described above. We further identified the functional role of *Wnt10b* in vitro by using lentivirus to infect the OP‐ASCs and found that overexpression of *Wnt10b* could upregulate β‐catenin and Lef1 to promote the ability of OP‐ASCs osteogenic differentiation, and suggests that *Wnt10b* regulates the OP‐ASCs' osteogenic capacity by the activation/inhibition of Wnt/β‐catenin signalling pathway. Moreover, we used composite scaffolds in combination with modified Wnt10b‐overexpressing OP‐ASCs and BCP to stimulate bone regeneration in the CSCD model of OP mice in vivo. Collectively, our results revealed that *Wnt10b* not only has a significant impact on promoting the regeneration of bone in vitro and in vivo experiments but may also act as a potential therapeutic application for OP patients with bone defects and other bone‐loss conditions.

## 2 MATERIALS AND METHODS

### 2.1 Animal ethics

The proposed research protocol for this project was approved by the Southwest Medical University's Ethics Committee (20180391222). This experimental operation was performed following the requirements of the Laboratory Animal Care and Usage Guidelines.

### 2.2 Establishment of an osteoporotic mice model and culture of OP‐ASCs and ASCs

Thirty female C57BL/6J mice aged 8 weeks from Teng Xin Biological Technology Co., Ltd., were randomly separated into two groups: the control (CON) group (*n* = 15) and the ovariectomy (OVX) group (*n* = 15). OP group mice were weighed and anaesthetised. Following the removal of back hair and disinfecting, a 0.5‐cm linear incision parallel to the spine was created at a distance of 0.5 cm from the spine and 1 cm below the ribs on the back of each mouse. Bilateral ovaries were separated and removed, and aseptic suturing of muscle and skin was performed. In the CON group, the identical procedure was done, but instead of bilateral ovaries, the same volume of adipose tissue was removed.^13^

As previously indicated,^25^ OP‐ASCs and ASCs were harvested and cultured. First, the inguinal adipose tissue was harvested from 14‐week‐old female OP and CON mice. Type I collagenase was used to digest the excised adipose tissue (Sigma‐Aldrich) for 20 min to obtain single cells. Then, the above cells were inoculated into culture flasks and incubated with α‐MEM medium (Alpha‐type modified Eagle's Medium) containing 1% penicillin/streptomycin and 10% fetal bovine serum (FBS). Every 3 days, the medium of cells was changed. Besides, all cells need to be passaged three times before following experiments.

Additionally, flow cytometry was performed to characterize the OP‐ASCs and ASCs in the study, and positive expression percentages of CD29, CD31 and CD45 were detected.^15^, ^26^

### 2.3 *Wnt10b* overexpression and interference

#### 2.3.1 Construction of *Wnt10b* overexpression lentivirus

The Wnt10b overexpression lentivirus was provided by OBiO Technology Co., Ltd. cDNA‐*Wnt10b* was synthesized following the standard DNA recombination methods. First, *Wnt10b* inserts were isolated from the pcDNA‐*Wnt10b* by PCR amplification. PCR products were sequenced to ensure the integrity of the entire *Wnt10b* coding sequence. Subsequently, the pLenti‐CMV‐EGFP‐3FLAG‐PGK‐Puro plasmids cloned with the *Wnt10b* inserts were transfected into 293T cells together with the packaging helper plasmids. The *Wnt10b* overexpression lentivirus (OE‐*Wnt10b*) was harvested from the filtered and concentrated supernatant of the *Wnt10b* virus. The mock viral supernatant (OE‐*Wnt10b*‐CON) was synthesized similarly, but without the *Wnt10b* insert. Antibiotic‐resistant cells were screened using 10 mg/mL puromycin for the follow‐up experiments.

#### 2.3.2 Construction of lentiviral vectors containing *Wnt10b* shRNA

The lentiviral vectors containing *Wnt10b* shRNA were also provided by OBiO Technology. Briefly, the oligonucleotides containing *Wnt10b* splice variant mRNA interference targets were prepared, annealed, and then ligated into the pLKD‐CMV‐G&PR‐U6‐shRNA lentiviral vector. The oligonucleotide sequences are 5′‐CCGGCGCTGCCTGGACAAGATCAATCTCGAGAT TGATCTTGTCCAGGCAGCGTTTTTTG‐3′ (sense) and 5′‐AATTCAAAAA ACGCTGCCTGGACAAGATCAATCTCGAGATTGATCTTGTCCAGGCAGCG‐3′ (antisense). The *Wnt10b* shRNA viral (KD‐*Wnt10b*) was prepared, filtered, concentrated and harvested as described above. The mock shRNA viral (KD‐*Wnt10b*‐NC), which did not contain the *Wnt10b* shRNA insert, was obtained similarly.

#### 2.3.3 Cell transfection

OP‐ASCs of passage 3 (P3) were inoculated into six‐well plates at a density of 5 × 10^4^ cells/well. OP‐ASCs were cultured and transfected with a lentivirus vector containing *Wnt10b* cDNA at different multiplicity of infection (MOI), including 0, 20, 40, 60, 80 and 100, in α‐MEM medium containing 1% penicillin/streptomycin and 10% FBS for 16 h. The appropriate MOI was confirmed according to the GFP‐positive proportion under a fluorescence microscope. OP‐ASCs were infected with different lentiviruses (OE‐*Wnt10b*, OE‐*Wnt10b*‐CON, KD‐*Wnt10b* and KD‐*Wnt10b*‐NC) at the appropriate MOI with 10 mg/mL polybrene for 16 h. At 7‐day post‐transfection, the RNA and protein expression of Wnt10b were examined by qPCR and WB assays.

### 2.4 Osteogenic induction and alizarin red staining

After transfection with various lentiviral vectors (OE‐*Wnt10b*, KD‐*Wnt10b* and Mock), these modified OP‐ASCs were inoculated in six‐well plates, then cultured in the osteogenic induction medium (Sigma‐Aldrich). On the 14th day of osteogenesis induction, the alizarin red staining was utilized to examine these cells' osteogenic capacities. Briefly, 4% paraformaldehyde was used to fix these modified OP‐ASCs for 30 min, followed by washing the cells three times with PBS, and 30 min at room temperature incubation with alizarin red dye. The mineralized nodules formed in each group were identified and videotaped under the bright‐field view of the inverted microscope, with at least three random images taken for each well.

### 2.5 qPCR analysis

First, the total RNA of all cells was extracted with Trizol (Invitrogen), successively purified, concentrated and finally detected by a spectrophotometer. According to the instructions of PrimeScript^RT^ Reagent Kit (TaKaRa), 500 ng of RNA was reverse transcription with a volume of 20 μL. As reported previously,^25^ Power SYBR Green PCR Master Mix (4367659) and the 7500 Rapid Real‐Time PCR System were used for qPCR. The relative mRNA expression levels were shown by the fold changes in comparison to the controls after normalization to the Gapdh. Table 1 lists the primers utilized in our investigation.

**TABLE 1:** Sequences of primers used for qPCR.

Primer	Sequence
Wnt10b	Forward: 5′‐TGTTCCTGGCTCAGTCCCCA‐3′ Reverse: 5′‐CCGCATTCTCGCCTGGATGT‐3′
β‐catenin	Forward: 5′‐TGGTGACAGGGAAGACATCA‐3′ Reverse: 5′‐CCACAACAGGCAGTCCATAA‐3′
Lef1	Forward: 5′‐ACGTTGCTCCTGTATAGACG‐3′ Reverse: 5′‐GCAGATATAGACACTAGCACC‐3′
Runx2	Forward: 5′‐CCGAACTGGTCCGCACCGAC‐3′ Reverse: 5′‐CTTGAAGGCCACGGGCAGGG‐3′
Opn	Forward: 5′‐GGATTCTGTGGACTCGGATG‐3′ Reverse: 5′‐CGACTGTAGGGACGATTGGA‐3′
Gapdh	Forward: 5′‐CTCGCTCCTGGAAGATGGTG‐3′ Reverse: 5′‐GGTGAAGGTCGGTGTGAACG‐3′

### 2.6 Western blot analysis

The acquisition of total proteins from cells was performed by the protein extraction kit (KeyGEN BioTECH), and the protein assay kit (TaKaRa) was used to detect concentration. Next, proteins were separated using an SDS‐PAGE gel and then transferred to the PVDF membranes. After being sealed with fat‐free milk for 1 h, the membranes were used in conjunction with primary antibodies against *Wnt10b* (ab70816), 3Flag(orb357990), β‐CATENIN (ab32572), LEF1 (ab137872), RUNX2 (ab92336), OPN (ab283656) and GAPDH (ab8245) overnight at 4°C. After that, secondary antibodies (ab288151) were used to interact with the primary antibodies. Immuno‐bands were developed by a Western blotting substrate for ECL and visualized with the ECL system. Finally, ImageJ software was performed to quantify the intensities of the individual immunoblots. The values of all samples were shown after normalization to the Gapdh.

### 2.7 Preparation of OP‐ASC‐seeded BCP scaffolds

Pie‐shaped (φ4.0 mm × 2.0 mm) porous BCP scaffolds with 50% porosity and an average pore diameter of 500 ± 45 μm were provided by Sichuan University Research Center. Before cell inoculation, BCP discs were autoclaved and then immersed in α‐MEM medium for 12 h. Then 0.1 mL OP‐ASCs cell suspension was seeded in BCP discs at a density of 1 × 10^6^ cells/mL and incubated for 4 h at 37°C before adding the culture medium.

### 2.8 The detection of cell proliferation

To investigate the adhesion and proliferation rate of OP‐ASCs to BCP scaffolds, the OP‐ASC‐seeded scaffolds were cocultured for 1, 3, 7 and 14 days, and subjected to scanning electron microscope (SEM; KYKY‐2800), CCK‐8 tests (Sigma‐Aldrich) and confocal laser scanning microscopy (Leica) separately.

Before conducting SEM detection, samples needed to be dried and sputter‐coated with gold. The absorbance of CCK‐8 was measured by a spectrophotometer at a wavelength of 450 nm. After the samples were stained with Phalloidin and DAPI according to the recommended protocol of the reagents, the detection of the confocal laser scanning microscope was performed. The samples were incubated with phalloidin (7 μg/mL, A22286) and DAPI solution (5 μg/mL, C1006).

### 2.9 In vivo implantation of OP‐ASCs‐seeded BCP composite scaffolds to the CSCD model

For the construction of the CSCD mice model, 72 OVX mice with bilateral ovaries removed for 6 weeks were randomly and equally divided into six groups of 12 mice each, and two 4‐mm diameter circular bone defects were created on the calvarium of these OP mice. Then, OP‐ASCs‐seeded BCP composite scaffolds were incubated in an osteogenic induction medium for 2 days, including (1) BCP scaffolds without cells, (2) BCP scaffolds with OP‐ASCs (Mock), (3) BCP scaffolds with OP‐ASCs overexpressing *Wnt10b* (OE‐*Wnt10b*), (4) BCP scaffolds with OP‐ASCs modified with control lentivirus overexpressing *Wnt10b* (OE‐*Wnt10b* CON), (5) BCP scaffolds with *Wnt10b* knockdown OP‐ASCs (KD‐*Wnt10b*) and (6) BCP scaffolds with OP‐ASCs modified with control lentivirus knockdown *Wnt10b* (KD‐*Wnt10b* NC) were transplanted into the defects. The incision was then closed with sutures. The mice could all function normally after the operation. There were 72 C57BL/6 mice (18–22 g) used for the study. There were three time points (4, 8 and 12 weeks) and six groups designed for the testing. Each time point contained 24 mice, with 4 mice and 8 defects for each group.

### 2.10 Micro‐computed tomography

The middle section of the right femur from the normal mice and OVX mice with bilateral ovariectomy for 6 weeks and the BCP scaffolds with/without modified OP‐ASCs transplanted into the CSCD for 4, 8 and 12 weeks were detected by micro‐computed tomography (micro‐CT; SIEMENS) in the scanning mode (voxel size, 10 mm; slice thickness, 18 mm; pixel matrix, 2048 × 2048) for the detection of bone volume. The three‐dimensional (3D) isosurface rendering software was applied to generate the 3D stereoscopic visualization. Mimics10.01 software was used to analyse the BMD, the percentages of new bone volume relative to tissue volume (BV/TV), the trabecular spacing (Tb.Sp) and the trabecular number (Tb.N).

### 2.11 Histochemical and immunohistochemical staining

After fixation in 4% paraformaldehyde for 48 h and decalcification with decalcifier solution for about 3 weeks, the femoral and calvarial bones from normal and OVX mice were infiltrated in paraffin and sliced into 10 μm slices, followed by hematoxylin and eosin (H&E) and Masson's trichrome staining.

For immunohistochemical staining, calvarial bone slices from OVX mice with implantation of composite scaffolds for 12 weeks were routinely deparaffinized and rehydrated, followed by antigen retrieval with trypsin at 37°C for 45 min. Slides were then incubated overnight at 4°C with Opn‐specific primary antibodies (1:200, ab63856). Subsequently, the secondary antibodies (1:1000, ab150077) were applied at room temperature for 1 h. Finally, cell nuclei were stained with haematoxylin.

### 2.12 Statistical analysis

Unless otherwise specified, all experiments were performed in triplicate. This experiment's data were reported as mean ± standard derivation (SD). Statistics were compared using an unpaired Student's *t* test, with *p <* 0.05 indicating statistical significance. SPSS 19.0 software was employed to examine all statistical data.

## 3 RESULTS

### 3.1 The osteoporotic mouse model was successfully constructed

The femur and proximal tibia obtained from CON and OVX mice at 6 weeks post‐operation were analysed by histopathological stainings such as H&E staining, Masson's trichrome staining, and micro‐CT scanning to confirm whether the osteoporotic model was successfully established.

Results for the histochemical staining are illustrated in Figure 1A. Compared with CON mice, OVX mice showed fewer and disordered or discontinuous bone trabeculae, thinner femoral cortical bone and enlarged bone marrow cavity, which indicates the occurrence of bone resorption and defect.

Micro‐CT results were similar to the histochemical staining analysis. Mice from the OP group presented thinner trabecular organization, reduced interconnectivity and increased separation compared with mice from the CON group (Figure 1B). Statistical analysis revealed that OP mice had higher Tb.Sp levels and lower Tb.N and BV/TV levels in comparison to CON mice. Based on these findings, we conclude that the osteoporotic mouse model was effectively developed.

### 3.2 The expression of *Wnt10b* RNA and protein in OP‐ASCs was decreased

At 6 weeks after surgery, OP‐ASCs and ASCs were extracted and cultivated from the inguinal adipose tissue of OVX and CON mice. After culture and passaging, both cell types appeared fibroblast‐like in shape and adopted polygon or spindle morphology as cell passage increased. In the experiments, we found that even if the two types of cells were continuously cultured to P3 and P4 (the data were not shown), there was no discernible variation in cell morphology (Figure 1C).

Wnt10b RNA and protein expression levels were measured using qPCR and WB in all cells. As shown in Figure 1D,E, the RNA expression of *Wnt10b* in OP‐ASCs was about 1/5 of that in ASCs, similarly, its protein expression was about 1/2 of that in ASCs. OP‐ASCs were found to exhibit lower *Wnt10b* expression than ASCs. These results verified that OP decreases the expression of *Wnt10b* in ASCs.

### 3.3 Knockdown of *Wnt10b* suppressed osteogenesis of OP‐ASCs in vitro

**FIGURE 2:**
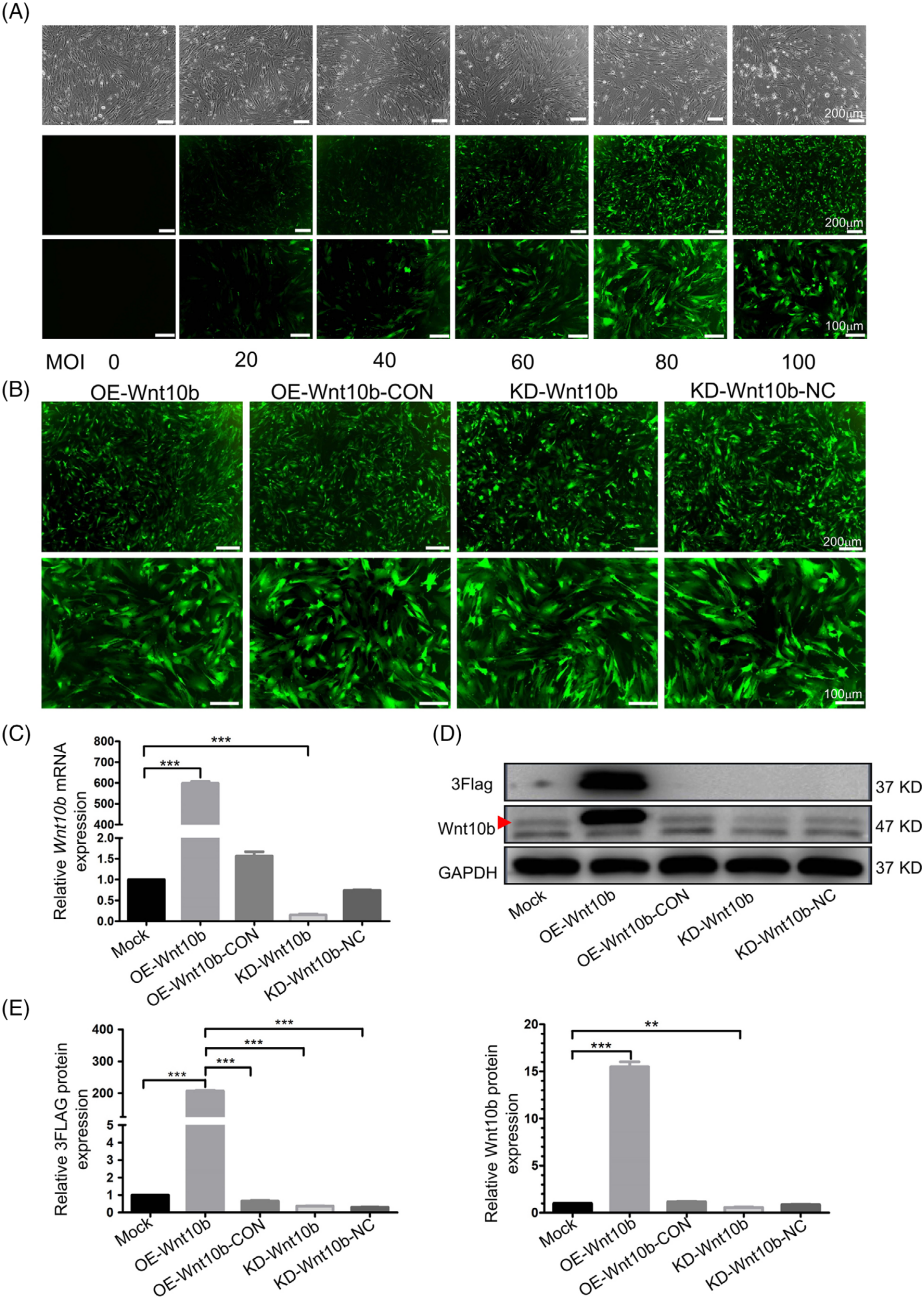
The expression of Wnt10b in OP‐ASCs could be effectively regulated by transfecting OP‐ASCs with different Wnt10b lentiviruses. (A) When the MOI = 80, the GFP fluorescence intensity in OP‐ASCs transfected with Lenti‐Wnt10b was the highest and maintained a good cell morphology. (B) The fluorescence expression rate of GFP in all OP‐ASCs transfected with four different lentiviruses was higher than 80% when MOI = 80. (C ) qPCR detection in OP‐ASCs transfected with four lentiviruses showed that OE‐Wnt10b/KD‐Wnt10b could significantly up/down‐regulate the gene expression of Wnt10b in OP‐ASCs (***p < 0.05), while OE‐Wnt10b‐CON/KD‐Wnt10b‐NC had no significant effect on the expression of Wnt10b gene. (D, E) The detection and quantitative analysis of WB in OP‐ASCs transfected with four lentiviruses, it was found that OE‐Wnt10b significantly increased the expression of Wnt10b protein and 3Flag (***p < 0.001), KD‐Wnt10b can significantly reduce the expression level of Wnt10b protein (**p < 0.01), while there was no significant change among OE‐Wnt10b‐CON/KD‐Wnt10b‐NC group compared with Mock group. Data represent the mean ± SD of at least three independent experiments. (*p < 0.05, **p < 0.01 and ***p < 0.001). MOI, multiplicity of infection; OP‐ASC, osteoporotic adipose‐derived stem cell.

**FIGURE 3:**
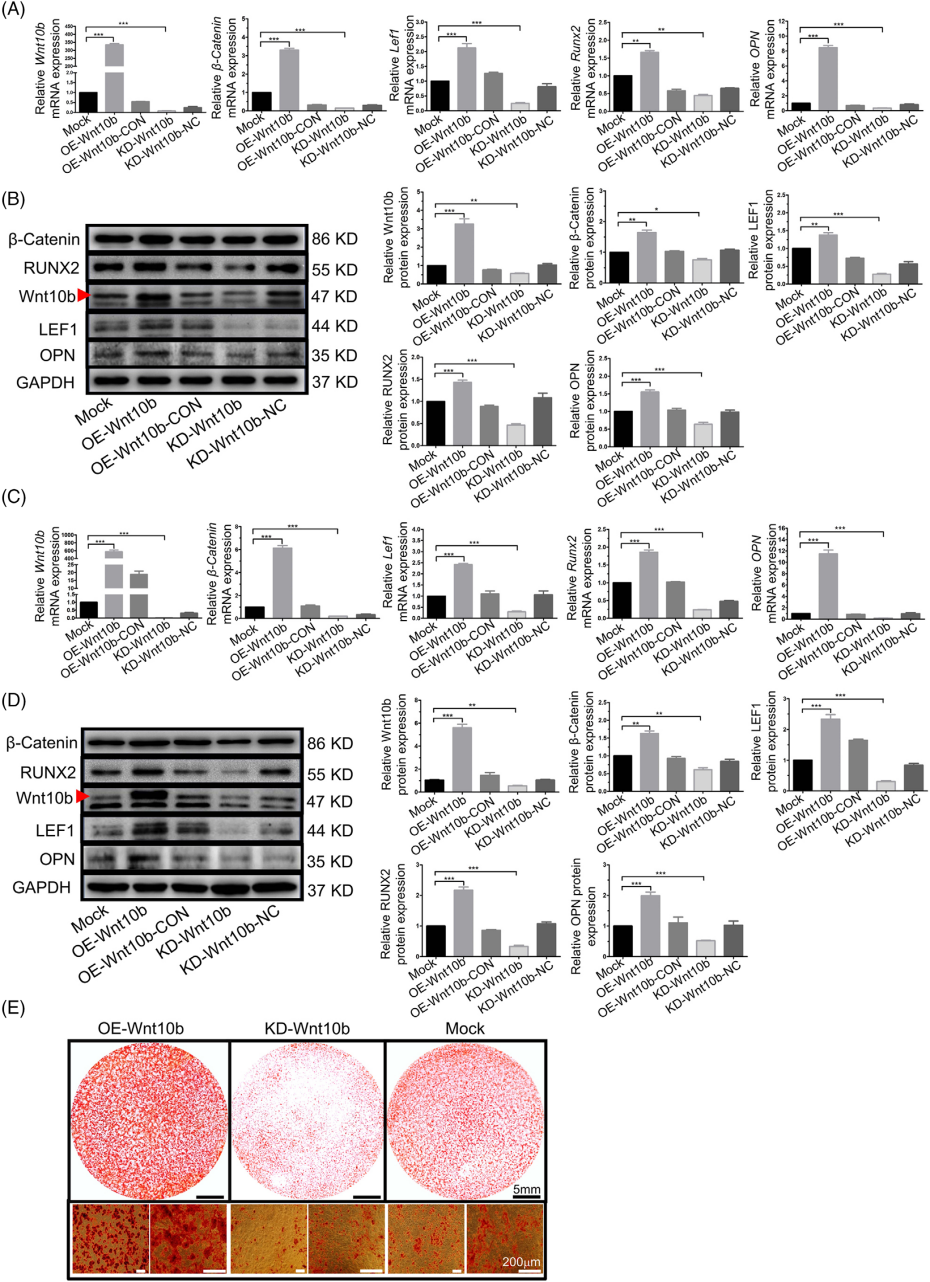
Overexpression of Wnt10b improved the osteogenesis ability of OP‐ASCs by activating canonical Wnt signalling pathway in vitro. (A, C) After Wnt10b transduction, the mRNA expression levels of β‐catenin, Lef1, Runx2 and OPN were greatly upregulated and downregulated by Lenti‐Wnt10b and Lenti‐shRNAWnt10b, respectively, on Days 3 and 7 of osteogenic induction (**p < 0.05 and ***p < 0.001). (B, D) The protein levels of β‐catenin, Lef1, Runx2 and OPN showed the same pattern as seen with mRNA expression (*p < 0.05, **p < 0.01 and ***p < 0.001). (E) Alizarin red staining on Day 14 shows the calcium deposition formed by the OE‐Wnt10b group was significantly more than the Mock group, but the calcium deposition that emerged in the KD‐Wnt10b group was the least among the three groups. Data represent the mean ± SD of at least three independent experiments (*p < 0.05, **p < 0.01 and ***p < 0.001). OP‐ASC, osteoporotic adipose‐derived stem cell; OPN, osteopontin.

To investigate the function of *Wnt10b* in OP‐ASCs, *Wnt10b* knockdown models (Figure 2A,B) in OP‐ASCs were established using lentiviruses constructed with *Wnt10b* shRNA sequences. The silencing efficacy showed that the levels of mRNA (Figure 2C) and protein (Figure 2D,E) of *Wnt10b* were successfully knocked down. After stable knockdown of *Wnt10b*, OP‐ASCs displayed decreased osteogenic differentiation ability. Specifically, after osteogenic induction for a period of 3 or 7 days, both mRNA and protein expression of factors in the canonical Wnt signalling pathway (β‐catenin, Lef1) and important osteogenic factors (Runx2, Opn) were decreased in *Wnt10b*‐knockdown OP‐ASCs (Figure 3A–D). Furthermore, the mineralized nodules formed in the *Wnt10b* knockdown OP‐ASCs were the least among the three groups after cultured in the osteogenic induction medium for 14 days (Figure 3E).

### 3.4 Overexpression of *Wnt10b* promoted osteogenesis of OP‐ASCs in vitro

The system of *Wnt10b* overexpression (Figure 2A,B) was also constructed using lentivirus to investigate the significance of *Wnt10b* in OP‐ASCs. The upregulation of *Wnt10b* in OP‐ASCs at both the RNA and protein levels (Figure 2C–E) was verified by transfecting the cells with *Wnt10b* overexpression lentivirus. After incubation in an osteogenic induction medium for 3 and 7 days, the expression levels of β‐catenin, Lef1, Runx2 and Opn in *Wnt10b*‐overexpressed OP‐ASCs were significantly elevated (Figure 3A–D). Moreover, after 14 days of cultivation in osteogenic induction medium, the modified OP‐ASCs in the OE‐*Wnt10b* group produced considerably more mineralized nodules compared to the other two groups (Figure 3F). The above results indicated that overexpression of *Wnt10b* might potentially improve the osteogenic potential of OP‐ASCs by the activation of the Wnt/β‐catenin signalling pathway in vitro.

### 3.5 BCP and OP‐ASCs combine to form a composite scaffold

**FIGURE 4:**
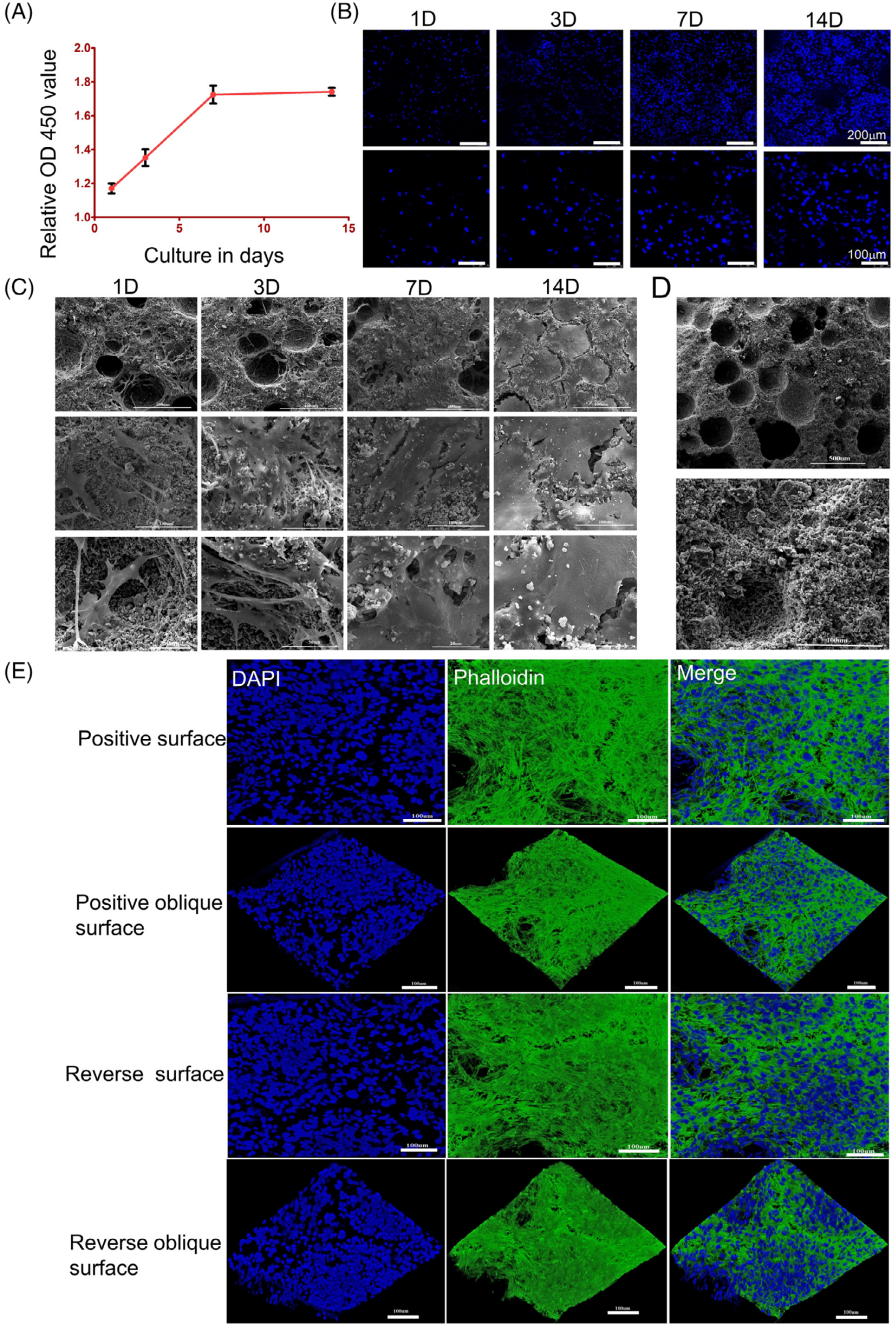
OP‐ASCs could seed and proliferate well with BCP. (A, B) CCK‐8 and DAPI staining show the number of OP‐ASCs increased with time. The cell proliferation rate was faster at 1, 3 and 7 days of coculture, while at 7–14 days, the proliferation rate was significantly slower. (C) SEM scanning shows the same proliferation pattern as the above expression and confirms that OP‐ASCs combined well into the BCP scaffolds. (D) The physical properties of BCP. (E) Confocal laser scanning microscopy revealed OP‐ASCs were intertwined into a network and grew to saturation at 14 days of co‐cultivation. BCP, biphasic calcium phosphate; OP‐ASC, osteoporotic adipose‐derived stem cell; SEM, scanning electron microscope.

To investigate the proliferation of OP‐ASCs on BCP scaffolds, CCK‐8 and DAPI staining assays were carried out at 1 day, 3, 7 and 14 days of co‐culture. We found that the number of OP‐ASCs increased with time (Figure 4A,B) and the rate of cell growth was accelerated at 1–7 days and significantly slowed at 7–14 days (Figure 4A).

Regarding the adhesion between OP‐ASCs and the BCP scaffold, SEM scanning showed that the BCP scaffold is three‐dimensionally porous with a rough surface and granular shape and that the OP‐ASCs grew into the pores and surface of the BCP scaffolds, resulting in adhesion of OP‐ASCs and BCP scaffolds (Figure 4C,D). The number of OP‐ASCs adhered to the BCP scaffold increased with time and the surface of the BCP scaffold was covered with abundant cell substrates secreted by OP‐ASCs when cultured for 7–14 days (Figure 4C).

Similarly, 3D reconstruction using confocal laser scanning microscopy revealed that OP‐ASCs were intertwined into a network and grew to saturation when co‐culture of BCP and OP‐ASCs occurred for 14 days (Figure 4E).

### 3.6 Knockdown of *Wnt10b* suppressed bone regeneration in vivo

**FIGURE 5:**
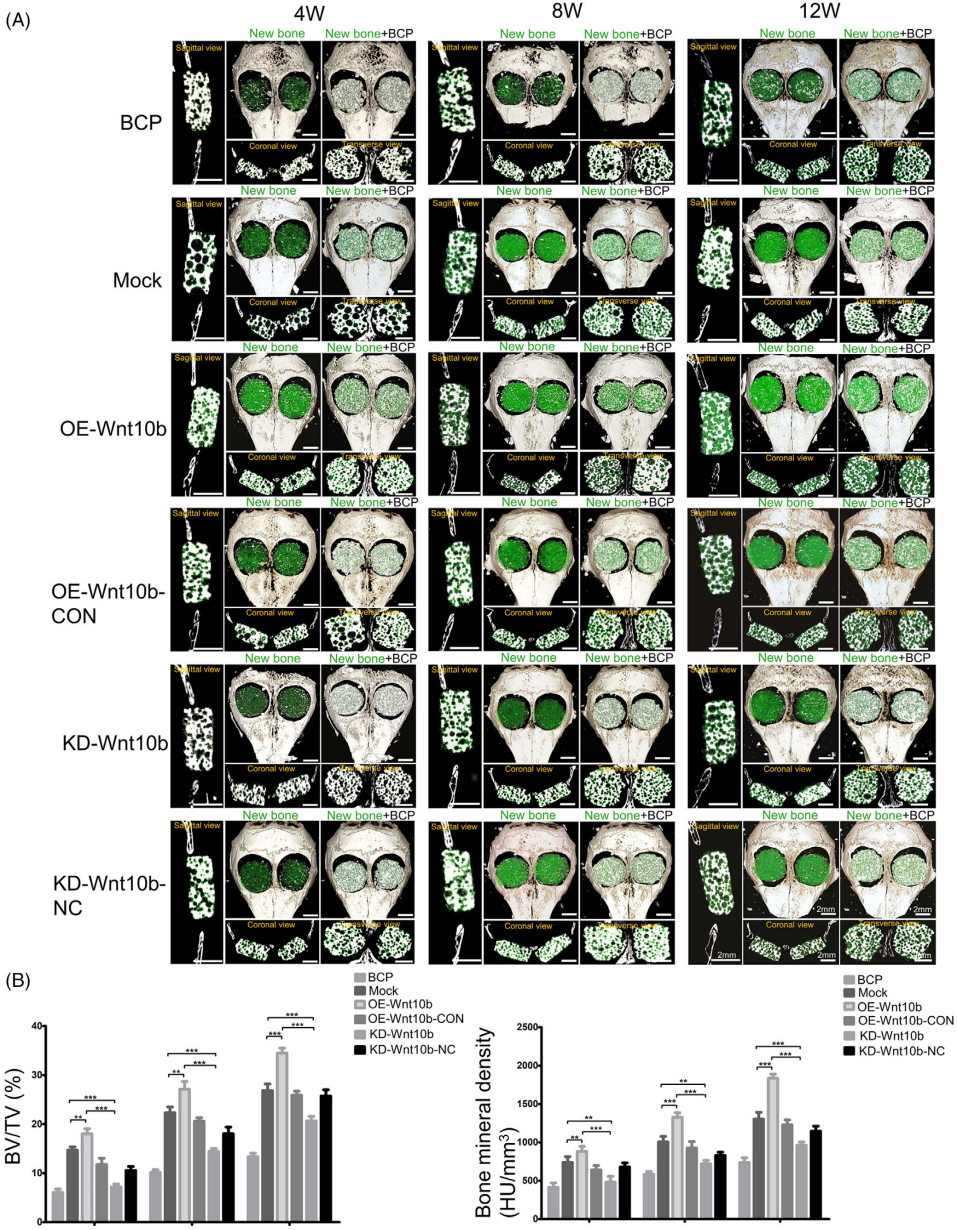
Overexpression of Wnt10b increased the amount of new bone formation to promote the repair of CSCD in osteoporotic mice in vivo. (A) Skulls from the mice implanted with modified OP‐ASCs were removed at 4 W (*n* = 24), 8 W (*n* = 24) and 12 W (*n* = 24) for micro‐CT analysis, the micro‐CT images showed the different reparative effects of the BCP, BCP with OP‐ASCs/Mock, BCP with OP‐ASCs/OE‐Wnt10b, BCP with OPASCs/OE‐Wnt10b‐CON, BCP with OP‐ASCs/KD‐Wnt10b and BCP with OP‐ASCs/ KD‐Wnt10b‐NC groups, as shown in sagittal view, coronal view, cross‐section and 3D surface rendering (green indicates new bone). (B) Bone volume/total volume (BV/TV) and bone mineral density (BMD) varied in every group at the three time points (**p < 0.001 and ***p < 0.0001). Data represent the mean ± SD of at least three independent experiments (*p < 0.05, **p < 0.001 and ***p < 0.001). BCP, biphasic calcium phosphate; CSCD, critical‐sized calvarial defect; micro‐CT, micro‐computed tomography; OP‐ASC, osteoporotic adipose‐derived stem cell; W, weeks.

**FIGURE 6:**
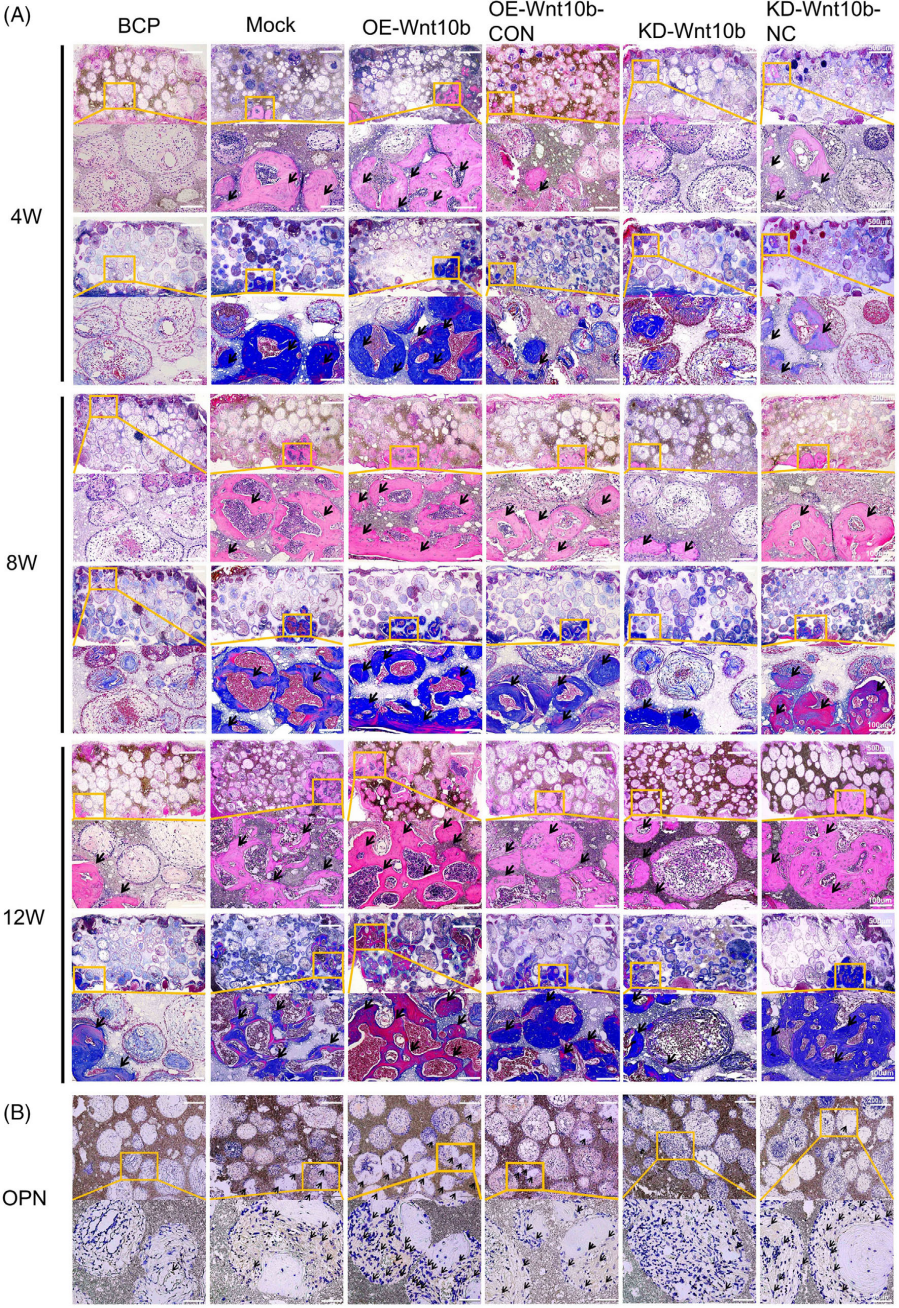
Overexpression of Wnt10b enhanced the osteogenic ability of OP‐ASCs in vivo. (A) H&E and Masson staining of composite scaffolds containing modified OP‐ASC and BCP in mice after 4/8/12 weeks of implantation. (B) Immunohistochemical staining specific for osteopontin (Opn) of composite scaffolds. Arrows indicate new bone in (A) and OPN deposition in (B), *n* = 3 for each group. BCP, biphasic calcium phosphate; H&E, haematoxylin and eosin; OP‐ASC, osteoporotic adipose‐derived stem cell.

To explore the functional role of *Wnt10b* in OP‐ASCs promoting bone generation in vivo, we transplanted composite scaffolds composed of modified OP‐ASCs transfected with the *Wnt10b* shRNA lentiviruses or the mock shRNA lentiviruses into CSCD of OP mice. After transplantation of the complexes for 4, 8 and 12 weeks, the condition of bone generation was analysed by micro‐CT, histochemical and immunohistochemical staining techniques. The results in Figure 5A,B illustrate that the amount of newly formed bone in composite scaffolds of each group showed an overall tendency to increase over time, but there are still significant differences among the groups (Figure 6A). Statistical analysis showed that the BMD of the KD‐*Wnt10b* group increased gradually over time (481.19 ± 131.72, 721.27 ± 76.86 and 962.28 ± 71.35 HU/cm^3^) and similar results were obtained with the percentages of BV/TV (7.19 ± 1.05%, 14.41 ± 1.06% and 20.66 ± 1.65%; Figure 6B). In addition, the amount of new bone formed in the composite scaffolds of the KD‐*Wnt10b* group was considerably less than that the group of KD‐*Wnt10b*‐NC at 4, 8 and 12 weeks. For the histochemical and immunohistochemical staining, as shown in Figure 6A, the amount of new bone in all groups also increased over time. Compared with the KD‐*Wnt10b*‐NC and BCP groups, the mineralized new bone content of the composite scaffolds of the KD‐*Wnt10b* group was the least at each period. Opn expression was detected at 12 weeks by immunohistochemical staining, with the KD‐*Wnt10b* group showing significantly lower Opn expression than the KD‐*Wnt10b*‐NC and BCP groups (Figure 6B).

### 3.7 Overexpression of *Wnt10b* promoted bone generation in vivo

OP‐ASCs–scaffold complexes composed of OP‐ASCs transfected with different lentiviruses, including *Wnt10b*‐overexpressed OP‐ASCs and mock‐infected cells, were transplanted into calvarial bone critical defect mice to further verify the osteogenesis‐promoting role of *Wnt10b* in vivo. The micro‐CT scan results revealed that the new bone's formation in all groups also increased over time (Figure 5A). The BMD of the new bone in scaffolds of OE‐*Wnt10b* group at 8 weeks after transplantation (1326.18 ± 107.59 HU/cm^3^) was lower than that at 12 weeks (1854 ± 117.40HU/cm^3^), but notably higher than that in 4 weeks (880.88 ± 117.40 HU/cm^3^) and (Figure 6B). The percentages of BV/TV in the OE‐*Wnt10b* group (18.44 ± 1.12% at 4 weeks, 27.46 ± 1.21% at 8 weeks and 35 ± 1.21% at 12 weeks) were similar to the results for BMD described above (Figure 5B). Additionally, the new bone formed of the OE‐*Wnt10b* group was notably more than that in the OE‐*Wnt10b*‐CON group at every point in time. We obtained similar results with the histological staining assay. From 4 to 12 weeks, the amount of mineralized new bone in all groups also showed a tendency to increase gradually over time (Figure 6A). It was found the OE‐Wnt10b group had the highest amount of new bone in each period compared with the OE‐*Wnt10b*‐CON and BCP groups (Figure 6A). Immunohistochemical staining revealed that the expression of Opn in the new bone of the OE‐Wnt10b group was greatest at 12 weeks, and substantially higher than the other two groups (Figure 6B).

Based on these results, we conclude that *Wnt10b* can promote the bone generation of OP‐ASCs in vivo.

## 4 DISCUSSION

As a common systemic bone disease, OP is characterized by the destruction of bone microstructure and a decrease in bone mass, which is prone to fracture.^24^, ^27^ OP is also a non‐communicable metabolic disease that is closely related to age and usually results from an imbalance between osteoblasts and osteoclasts due to postmenopausal oestrogen deficiency.^28^ Oestrogen deficiency may lead to OP by decreasing the strength, volume, and mineral density of bone and enhancing bone turnover, resulting in fractures and bone defects.^29^ This leads to an increased risk of fracture, prolonged bone defect healing time, decreased formation of callus, and deteriorated biomechanical properties of new bone. Despite efforts to focus on fracture prevention and the development of treatments to enhance bone mass and BMD, osteoporotic bone regeneration has received less attention.^30^ Compared with conventional methods, combining autologous modified stem cells and composite scaffolds may be more effective in repairing osteoporotic bone defects.^31^, ^32^

OVX mice have been regarded as a suitable animal model for studying OP‐related bone diseases because the pathological changes in these mice resemble those seen in postmenopausal OP.^33^ OP results from a functional imbalance between osteoblasts and osteoclasts.^34^ The CSCD model is often used to study the repair of bone defects because it can offer an appropriate analogue for the common scenarios observed in the clinic.^35^ To enhance bone tissue regeneration and reverse the imbalance in patients with OP, we transplanted OP‐ASCs overexpressing *Wnt10b* and BCP scaffold complexes into the CSCD of OVX mice to explore their effects on bone regeneration. As far as we know, this report is the first to apply a composite scaffold material of Wnt10b‐modified OP‐ASCs combined with BCP to repair CSCD repair in OVX osteoporotic mice.

As we know, the repair of bone defects in osteoporotic patients has always been a great challenge for physicians.^2^, ^36^ Current treatment approaches to osteoporotic bone defects are not always satisfactory. Several investigations have revealed that tissue engineering technologies provide a new therapeutic strategy for repairing tissue defects.^37^, ^38^, ^39^ The approaches of gene therapy to improve bone tissue regeneration are considered one of the most fascinating topics of tissue engineering.^40^, ^41^ Utilizing a tissue‐engineering material combination of autologous OP‐ASCs of gene‐modified and composite scaffolds to heal OP bone defects can overcome several limitations of existing approaches.^15^ This study demonstrated that *Wnt10b* significantly enhanced OP‐ASC osteogenesis by triggering the Wnt/β‐catenin signalling pathway, and indicated that *Wnt10b* holds an important position in the osteogenic differentiation and bone regenerative capacity of OP‐ASCs via modulating Runx2 and Opn expression. *Wnt10b*‐overexpressed OP‐ASCs dramatically improved the repair of the CSCD of ovariectomized osteoporotic mice in vivo.

ASCs are favoured by a wide range of researchers because of their easily available and abundant content. Compared with BMSCs, ASCs are still easier to cultivate and preserve, grow faster, and are more resistant,^42^, ^43^ which has made them a point of interest in recent research. In the study, we discovered that the OP‐ASCs' osteogenic differentiation capabilities, as measured by Runx2 and Opn mRNA and protein expression, as well as the number of mineralized nodules generated, were much lower than that of ASCs from normal mice.

The Wnt signalling pathway is regarded as an important pathway to regulate bone balance in osteoporotic mice.^44^, ^45^ *Wnt10b*, a bone anabolic Wnt ligand, is an important endogenous factor in the Wnt signalling pathway that regulates bone formation. The regulatory mechanisms of *Wnt10b* include inducing the differentiation of stem cells towards the phenotype of osteoblasts by promoting the expression of the factors associated with osteogenesis including Runx2, osterix and Dlx5, and inhibiting the expression of PPARγ and C/EBPα to restrain the differentiation of stem cell towards the adipocytic lineage.^17^, ^18^, ^46^, ^47^ Studies on transgenic mice showed that the upregulated expression of *Wnt10b* coincided with an increased bone mass and the above effect was mainly attributed to enhanced osteoblastogenesis.^17^ Some researchers have shown that *Wnt10b* could increase bone mass in OP by promoting T cell *Wnt10b* production and inhibiting CD28 costimulation by CTLA‐4Ig.^48^ Some studies showed that loading could promote osteogenesis and increase many Wnt signalling pathway‐related factors' expression by the upregulation of the expression of *Wnt10b*, thus indicating its important role in mechanical transduction to increase the formation of bone.^49^, ^50^ In our study, it was found that compared with ASCs, the *Wnt10b* expression of OP‐ASCs was significantly reduced at both the RNA and protein levels, while the OP‐ASCs' osteogenic capabilities were also significantly impaired. Therefore, we speculate that the impaired osteogenic capability of OP‐ASCs may be related to the decreased expression of *Wnt10b*. However, there is little known about the specific mechanism of *Wnt10b* that may modulate osteogenesis in OP. We further found that the gene and protein levels of osteogenesis‐specific markers (Runx2 and Opn) and the factors in Wnt/β‐catenin signalling pathway factors (β‐catenin and Lef1) were increased when OP‐ASCs were transfected with *Wnt10b* overexpression lentivirus and decreased when transfected with lentivirus that interfered with *Wnt10b*. Moreover, overexpression of *Wnt10b* promoted the generation of mineralized nodules in OP‐ASCs, while inhibition of *Wnt10* expression reduced the number of mineralized nodules. These results suggest that *Wnt10b* may enhance the osteotropic differentiation capacity of OP‐ASCs with activation of the canonical Wnt signalling pathway by increasing the β‐catenin expression and accelerating its entrance into the nucleus. Subsequently, β‐catenin stimulated the expression of osteogenic‐target factors (Runx2 and Lef1), then ultimately promoted the OP‐ASCs' osteogenic ability to repair bone defects.

The functional role of *Wnt10b* in bone tissue regeneration was evaluated by repairing the CSCD in the osteoporotic mouse model. At 4, 8 and 12 weeks, 3D reconstruction data of micro‐CT indicated that overexpression of *Wnt10b* through the application of OP‐ASCs significantly improved ossification in the CSCD mice model. On the contrary, just less new bone formed was found in both BCP and KD‐*Wnt10b* groups from 4 to 12 weeks. Thus, *Wnt10b* can efficiently improve the OP‐ASCs' osteogenic ability to generate more new bone in vivo. In a word, the data of the study suggested that *Wnt10b* could activate the canonical Wnt pathway, and would become a potential target to promote bone tissue regeneration by osteoinductive OP‐ASCs and acts to increase the osteogenesis of OP‐ASCs.

BCP ceramics are a mixture formed by β‐TCP and HA in different proportions and have been commercially available and are increasingly accepted as bone repair materials in many medical and dental applications. Many studies have indicated that the BCP scaffolds can promote bone tissue regeneration.^51^, ^52^ In addition, the biological activity of BCP can also be improved by optimizing physicochemical properties to promote bone regeneration. Researchers have reported that the ratio of 70/30 (HA/TCP) showed more favourable and faster bone resorption in terms of space maintenance and new bone formation compared to the 80/20 ratio.^53^ Hung et al.^54^ had been reported that the ratios of 60/40 and 70/30 showed a better bone regeneration rate than other groups and without any inflammatory response and toxic effects. Therefore, we chose BCP (HA/TCP = 70/30) combined with OP‐ASCs and found that the number of cells growing on the scaffolds increased over time as evaluated by CCK‐8, DAPI and SEM scanning. When co‐cultured for 1–7 days, the proliferation rate of OP‐ASCs showing a gradual increase, while there was a decrease at 7–14 days, which may be related to the saturation of cell growth. Furthermore, similar results were found in vivo experiments, where the amount of new bone formed in the scaffolds increased over time in each group, and no inflammation or toxic side effects were observed. These results showed that BCP had good biocompatibility without cytotoxicity, and combined well with OP‐ASCs to promote cell proliferation.

## 5 CONCLUSION

In summary, we have demonstrated that overexpressing *Wnt10b* can activate the Wnt/β‐catenin signalling pathway to boost the differentiation of OP‐ASCs towards osteogenesis, reverse the impaired osteogenic differentiation capabilities of OP‐ASCs, and then enhance the repair of bone defects in osteoporotic mice. Our findings show that *Wnt10b* plays a significant functional role in the osteogenic differentiation of OP‐ASCs and provide preclinical evidence for the potential applications of *Wnt10b*‐engineered OP‐ASCs to stimulate bone repair and regeneration in OP patients.

## AUTHOR CONTRIBUTIONS

Kui Huang established osteoporotic mouse models, performed in vitro and in vivo studies, analyzed and organized experimental data, and drafted the article. Shuyu Cai, Ting Fu, Qiang Zhu and Lin Liu performed the harvest and culture of cells, collection and analysis of experimental data. Zhihao Yao and Pengcheng Rao collected and analyzed experimental data. Xiaorong Lan performed data analysis, paper revision and funding procurement. Qing Li performed project supervision and manuscript editing. Jingang Xiao conceived the idea, developed the experimental study, collected data, analyzed it, reviewed the article and offered financial assistance. The final paper has been reviewed and approved by all writers.

## CONFLICT OF INTEREST STATEMENT

The authors declare no conflicts of interest.

## Data Availability

The data in the article are available to be obtained from the corresponding author upon reasonable request.

## References

[1] Ensrud KE , Crandall CJ . Osteoporosis. Ann Intern Med. 2017;167(3):ITC17‐ITC32. 28761958 10.7326/AITC201708010

[2] Wagner JM , Conze N , Lewik G , et al. Bone allografts combined with adipose‐derived stem cells in an optimized cell/volume ratio showed enhanced osteogenesis and angiogenesis in a murine femur defect model. J Mol Med. 2019;97(10):1439‐1450. 31367858 10.1007/s00109-019-01822-9

[3] McMillan A , Nguyen MK , Gonzalez‐Fernandez T , et al. Dual non‐viral gene delivery from microparticles within 3D high‐density stem cell constructs for enhanced bone tissue engineering. Biomaterials. 2018;161:240‐255. 29421560 10.1016/j.biomaterials.2018.01.006 PMC5826638

[4] Fu N , Meng Z , Jiao T , et al. Radial P34HB electrospun fiber: a scaffold for bone tissue engineering. J Nanosci Nanotechnol. 2020;20(10):6161‐6167. 32384966 10.1166/jnn.2020.18583

[5] Zhang Y , Ma W , Zhan Y , et al. Nucleic acids and analogs for bone regeneration. Bone Res. 2018;6:37. 30603226 10.1038/s41413-018-0042-7 PMC6306486

[6] Fan J , Im CS , Guo M , et al. Enhanced osteogenesis of adipose‐derived stem cells by regulating bone morphogenetic protein signaling antagonists and agonists. Stem Cells Transl Med. 2016;5(4):539‐551. 26956209 10.5966/sctm.2015-0249 PMC4798741

[7] Yoshikawa M , Nakasa T , Ishikawa M , Adachi N , Ochi M . Evaluation of autologous skeletal muscle‐derived factors for regenerative medicine applications. Bone Joint Res. 2017;6(5):277‐283. 28473335 10.1302/2046-3758.65.BJR-2016-0187.R1 PMC5457645

[8] Clark AY , Martin KE , García JR , et al. Integrin‐specific hydrogels modulate transplanted human bone marrow‐derived mesenchymal stem cell survival, engraftment, and reparative activities. Nat Commun. 2020;11(1):114. 31913286 10.1038/s41467-019-14000-9 PMC6949269

[9] Li W , Liu Y , Zhang P , et al. Tissue‐engineered bone immobilized with human adipose stem cells‐derived exosomes promotes bone regeneration. ACS Appl Mater Interfaces. 2018;10(6):5240‐5254. 29359912 10.1021/acsami.7b17620

[10] Li Y , Wang L , Zhang M , et al. Advanced glycation end products inhibit the osteogenic differentiation potential of adipose‐derived stem cells by modulating Wnt/β‐catenin signaling pathway via DNA methylation. Cell Prolif. 2020;53(6):e12834. 32468637 10.1111/cpr.12834 PMC7309593

[11] Zhang M , Li Y , Rao P , et al. Blockade of receptors of advanced glycation end products ameliorates diabetic osteogenesis of adipose‐derived stem cells through DNA methylation and Wnt signaling pathway. Cell Prolif. 2018;51(5):e12471. 30014569 10.1111/cpr.12471 PMC6528890

[12] Xiao J , Yang X , Jing W , et al. Adipogenic and osteogenic differentiation of Lin(−)CD271(+)Sca‐1(+) adipose‐derived stem cells. Mol Cell Biochem. 2013;377(1‐2):107‐119. 23430356 10.1007/s11010-013-1575-0

[13] Xie Q , Xie J , Zhong J , et al. Hypoxia enhances angiogenesis in an adipose‐derived stromal cell/endothelial cell co‐culture 3D gel model. Cell Prolif. 2016;49(2):236‐245. 26997164 10.1111/cpr.12244 PMC6496300

[14] Zhou W , Lin J , Zhao K , et al. Single‐cell profiles and clinically useful properties of human mesenchymal stem cells of adipose and bone marrow origin. Am J Sports Med. 2019;47(7):1722‐1733. 31100005 10.1177/0363546519848678

[15] Wang L , Huang C , Li Q , et al. Osteogenic differentiation potential of adipose‐derived stem cells from ovariectomized mice. Cell Prolif. 2017;50(2):e12328. 28090705 10.1111/cpr.12328 PMC6529141

[16] Peng S , Gao Y , Shi S , et al. LncRNA‐AK137033 inhibits the osteogenic potential of adipose‐derived stem cells in diabetic osteoporosis by regulating Wnt signaling pathway via DNA methylation. Cell Prolif. 2022;55(1):e13174. 34953002 10.1111/cpr.13174 PMC8780896

[17] Wu T , Tang H , Yang J , et al. METTL3‐m6 A methylase regulates the osteogenic potential of bone marrow mesenchymal stem cells in osteoporotic rats via the Wnt signaling pathway. Cell Prolif. 2022;55(5):e13234. 35470497 10.1111/cpr.13234 PMC9136513

[18] Bennett CN , Ouyang H , Ma YL , et al. Wnt10b increases postnatal bone formation by enhancing osteoblast differentiation. J Bone Miner Res. 2007;22(12):1924‐1932. 17708715 10.1359/jbmr.070810

[19] Cawthorn WP , Bree AJ , Yao Y , et al. Wnt6, Wnt10a and Wnt10b inhibit adipogenesis and stimulate osteoblastogenesis through a β‐catenin‐dependent mechanism. Bone. 2012;50(2):477‐489. 21872687 10.1016/j.bone.2011.08.010 PMC3261372

[20] Bennett CN , Longo KA , Wright WS , et al. Regulation of osteoblastogenesis and bone mass by Wnt10b. Proc Natl Acad Sci U S A. 2005;102(9):3324‐3329. 15728361 10.1073/pnas.0408742102 PMC552924

[21] Collins FL , Rios‐Arce ND , McCabe LR , et al. Cytokine and hormonal regulation of bone marrow immune cell Wnt10b expression. PLoS One. 2017;12(8):0181979. 10.1371/journal.pone.0181979 PMC5553813 28800644

[22] Hsu MN , Huang KL , Yu FJ , et al. Coactivation of endogenous Wnt10b and Foxc2 by CRISPR activation enhances BMSC osteogenesis and promotes calvarial bone regeneration. Mol Ther. 2020;28(2):441‐451. 31882321 10.1016/j.ymthe.2019.11.029 PMC7001053

[23] Bouler JM , Pilet P , Gauthier O , Verron E . Biphasic calcium phosphate ceramics for bone reconstruction: a review of biological response. Acta Biomater. 2017;53:1‐12. 28159720 10.1016/j.actbio.2017.01.076

[24] Ebrahimi M , Botelho MG , Dorozhkin SV . Biphasic calcium phosphates bioceramics (HA/TCP): concept, physicochemical properties and the impact of standardization of study protocols in biomaterials research. Mater Sci Eng C Mater Biol Appl. 2017;71:1293‐1312. 27987685 10.1016/j.msec.2016.11.039

[25] Wang FD , Wu PF , Chen SJ . Distribution of virulence genes in bacteremic methicillin‐resistant *Staphylococcus aureus* isolates from various sources. J Microbiol Immunol Infect. 2019;52(3):426‐432. 30686615 10.1016/j.jmii.2019.01.001

[26] Zheng CX , Sui BD , Liu N , et al. Adipose mesenchymal stem cells from osteoporotic donors preserve functionality and modulate systemic inflammatory microenvironment in osteoporotic cytotherapy. Sci Rep. 2018;8(1):5215. 29581449 10.1038/s41598-018-23098-8 PMC5980002

[27] Paspaliaris V , Kolios G . Stem cells in osteoporosis: from biology to new therapeutic approaches. Stem Cells Int. 2019;2019:1730978. 31281368 10.1155/2019/1730978 PMC6589256

[28] Cheng CH , Chen LR , Chen KH . Osteoporosis due to hormone imbalance: an overview of the effects of estrogen deficiency and glucocorticoid overuse on bone turnover. Int J Mol Sci. 2022;23(3):1376. 35163300 10.3390/ijms23031376 PMC8836058

[29] Pacifici R . Estrogen, cytokines, and pathogenesis of postmenopausal osteoporosis. J Bone Miner Res. 1996;11(8):1043‐1051. 8854239 10.1002/jbmr.5650110802

[30] Cun X , Xie J , Lin S , et al. Gene profile of soluble growth factors involved in angiogenesis, in an adipose‐derived stromal cell/endothelial cell co‐culture, 3D gel model. Cell Prolif. 2015;48(4):405‐412. 26037311 10.1111/cpr.12193 PMC6496362

[31] Huang K , Li Q , Li Y , et al. Cartilage tissue regeneration: the roles of cells, stimulating factors and scaffolds. Curr Stem Cell Res Ther. 2018;13(7):547‐567. 28595567 10.2174/1574888X12666170608080722

[32] Wang L , Li Y , Zhang M , Huang K , Peng S , Xiao J . Application of nanomaterials in regulating the fate of adipose‐derived stem cells. Curr Stem Cell Res Ther. 2021;16(1):3‐13. 32357820 10.2174/1574888X15666200502000343

[33] Li J , Li X , Liu D , et al. eIF2α signaling regulates autophagy of osteoblasts and the development of osteoclasts in OVX mice. Cell Death Dis. 2019;10(12):921. 31801950 10.1038/s41419-019-2159-z PMC6892793

[34] Wang S , Deng Z , Ma Y , et al. The role of autophagy and mitophagy in bone metabolic disorders. Int J Biol Sci. 2020;16(14):2675‐2691. 32792864 10.7150/ijbs.46627 PMC7415419

[35] Gao X , Hwang MP , Wright N , et al. The use of heparin/polycation coacervate sustain release system to compare the bone regenerative potentials of 5 BMPs using a critical sized calvarial bone defect model. Biomaterials. 2022;288:121708. 36031459 10.1016/j.biomaterials.2022.121708 PMC10129760

[36] Bover J , Bailone L , López‐Báez V , et al. Osteoporosis, bone mineral density and CKD‐MBD: treatment considerations. J Nephrol. 2017;30(5):677‐687. 28432640 10.1007/s40620-017-0404-z

[37] Wang L , Qiu Y , Guo Y , et al. Smart, elastic, and nanofiber‐based 3D scaffolds with self‐deploying capability for osteoporotic bone regeneration. Nano Lett. 2019;19(12):9112‐9120. 31765166 10.1021/acs.nanolett.9b04313

[38] Zhang T , Tian T , Lin Y . Functionalizing framework nucleic‐acid‐based nanostructures for biomedical application. Adv Mater. 2022;34(46):e2107820. 34787933 10.1002/adma.202107820

[39] Wei JQ , Liu Y , Zhang XH , et al. Enhanced critical‐sized bone defect repair efficiency by combining deproteinized antler cancellous bone and autologous BMSCs. Chin Chem Lett. 2017;28:845‐850.

[40] Qi H , Xu Y , Hu P , Yao C , Yang D . Construction and applications of DNA‐based nanomaterials in cancer therapy. Chin Chem Lett. 2022;33(3):1131‐1140.

[41] Chen X , Yang H , Song X , et al. Integrated and dual‐responsive lipopeptide nanovector with parallel effect to tumor and micro‐environment regulation by efficient gene and drug co‐delivery. Chin Chem Lett. 2022;24:107753.

[42] Tevlin R , desJardins‐Park H , Huber J , et al. Musculoskeletal tissue engineering: adipose derived stromal cell implementation for the treatment of osteoarthritis. Biomaterials. 2022;286:121544. 35633592 10.1016/j.biomaterials.2022.121544 PMC9267037

[43] Bacakova L , Zarubova J , Travnickova M , et al. Stem cells: their source, potency and use in regenerative therapies with focus on adipose‐derived stem cells—a review. Biotechnol Adv. 2018;36(4):1111‐1126. 29563048 10.1016/j.biotechadv.2018.03.011

[44] Liu J , Xiao Q , Xiao J , et al. Wnt/β‐catenin signalling: function, biological mechanisms, and therapeutic opportunities. Signal Transduct Target Ther. 2022;7(1):3. 34980884 10.1038/s41392-021-00762-6 PMC8724284

[45] Kim P , Park J , Lee DJ , et al. Mast4 determines the cell fate of MSCs for bone and cartilage development. Nat Commun. 2022;13(1):3960. 35803931 10.1038/s41467-022-31697-3 PMC9270402

[46] Hu Y , He Y , Fang J , et al. Wnt10b‐overexpressing umbilical cord mesenchymal stem cells promote fracture healing via accelerated cartilage callus to bone remodeling. Bioengineered. 2022;13(4):10313‐10323. 35436412 10.1080/21655979.2022.2062954 PMC9161882

[47] Liu H , Zhang N , Liu Y , Liu L , Yin G , Luo E . Effect of human Wnt10b transgene overexpression on peri‐implant osteogenesis in ovariectomized rats. Hum Gene Ther. 2018;29(12):1416‐1427. 29790378 10.1089/hum.2018.003 PMC12199630

[48] Roser‐Page S , Vikulina T , Zayzafoon M , Weitzmann MN . CTLA‐4Ig‐induced T cell anergy promotes Wnt‐10b production and bone formation in a mouse model. Arthritis Rheumatol. 2014;66(4):990‐999. 24757150 10.1002/art.38319 PMC3994890

[49] Komori T . Regulation of proliferation, differentiation and functions of osteoblasts by Runx2. Int J Mol Sci. 2019;20(7):1694. 30987410 10.3390/ijms20071694 PMC6480215

[50] Hou WW , Zhu ZL , Zhou Y , Zhang CX , Yu HY . Involvement of Wnt activation in the micromechanical vibration‐enhanced osteogenic response of osteoblasts. J Orthop Sci. 2011;16(5):598‐605. 21833614 10.1007/s00776-011-0124-5

[51] Li X , Song T , Chen X , et al. Osteoinductivity of porous biphasic calcium phosphate ceramic spheres with nanocrystalline and their efficacy in guiding bone regeneration. ACS Appl Mater Interfaces. 2019;11(4):3722‐3736. 30629405 10.1021/acsami.8b18525

[52] Gan D , Liu M , Xu T , Wang K , Tan H , Lu X . Chitosan/biphasic calcium phosphate scaffolds functionalized with BMP‐2‐encapsulated nanoparticles and RGD for bone regeneration. J Biomed Mater Res A. 2018;106(10):2613‐2624. 29790251 10.1002/jbm.a.36453

[53] Yun PY , Kim YK , Jeong KI , Park JC , Choi YJ . Influence of bone morphogenetic protein and proportion of hydroxyapatite on new bone formation in biphasic calcium phosphate graft: two pilot studies in animal bony defect model. J Craniomaxillofac Surg. 2014;42(8):1909‐1917. 25443868 10.1016/j.jcms.2014.07.011

[54] Hung CL , Yang JC , Chang WJ , et al. In vivo graft performance of an improved bone substitute composed of poor crystalline hydroxyapatite based biphasic calcium phosphate. Dent Mater J. 2011;30(1):21‐28. 21282892 10.4012/dmj.2010-060

